# Single-cell analysis of a tumor-derived exosome signature correlates with prognosis and immunotherapy response

**DOI:** 10.1186/s12967-021-03053-4

**Published:** 2021-09-08

**Authors:** Jiani Wu, Dongqiang Zeng, Shimeng Zhi, Zilan Ye, Wenjun Qiu, Na Huang, Li Sun, Chunlin Wang, Zhenzhen Wu, Jianping Bin, Yulin Liao, Min Shi, Wangjun Liao

**Affiliations:** 1 Department of Oncology, Nanfang Hospital, Southern Medical University, Guangzhou, Guangdong People’s Republic of China; 2 Department of Cardiology, State Key Laboratory of Organ Failure Research, Nanfang Hospital, Southern Medical University, Guangzhou, Guangdong People’s Republic of China

**Keywords:** Tumor-derived exosome, Single-cell analysis, Biomarker, Immunotherapy, Tumor microenvironment

## Abstract

**Background:**

Tumor-derived exosomes (TEXs) are involved in tumor progression and the immune modulation process and mediate intercellular communication in the tumor microenvironment. Although exosomes are considered promising liquid biomarkers for disease diagnosis, it is difficult to discriminate TEXs and to develop TEX-based predictive biomarkers.

**Methods:**

In this study, the gene expression profiles and clinical information were collected from The Cancer Genome Atlas (TCGA) database, IMvigor210 cohorts, and six independent Gene Expression Omnibus datasets. A TEXs-associated signature named TEXscore was established to predict overall survival in multiple cancer types and in patients undergoing immune checkpoint blockade therapies.

**Results:**

Based on exosome-associated genes, we first constructed a tumor-derived exosome signature named TEXscore using a principal component analysis algorithm. In single-cell RNA-sequencing data analysis, ascending TEXscore was associated with disease progression and poor clinical outcomes. In the TCGA Pan-Cancer cohort, TEXscore was elevated in tumor samples rather than in normal tissues, thereby serving as a reliable biomarker to distinguish cancer from non-cancer sources. Moreover, high TEXscore was associated with shorter overall survival across 12 cancer types. TEXscore showed great potential in predicting immunotherapy response in melanoma, urothelial cancer, and renal cancer. The immunosuppressive microenvironment characterized by macrophages, cancer-associated fibroblasts, and myeloid-derived suppressor cells was associated with high TEXscore in the TCGA and immunotherapy cohorts. Besides, TEXscore-associated miRNAs and gene mutations were also identified. Further experimental research will facilitate the extending of TEXscore in tumor-associated exosomes.

**Conclusions:**

TEXscore capturing tumor-derived exosome features might be a robust biomarker for prognosis and treatment responses in independent cohorts.

**Supplementary Information:**

The online version contains supplementary material available at 10.1186/s12967-021-03053-4.

## Introduction

Exosomes are 30–150 nm-sized extracellular vesicles of endosomal origin that inherit constituents from multiple cell types [1]. Relying on the plentiful cargos including proteins, nucleic acids, lipids, and metabolites, exosomes participate in intercellular communication and further influence the surrounding microenvironment in both biological and pathological conditions [2, 3]. Although all cell types are capable of generating exosomes, tumors are especially prone to massively releasing exosomes. The level of exosomes in plasma or other body fluids of patients with tumors is higher than that in normal donors [4]. Increasing evidence indicates that tumor-derived exosomes (TEXs) emerge as a vital modulator in various tumorigenesis processes, including tumor invasion, metastasis, and treatment resistance [5]. TEXs are taken up by immune cells and stromal cells that constitute the tumor microenvironment (TME), thereby changing TME infiltration pattern and affecting cell behavior [6, 7].

The unique message carried in exosomes, their abundance in peripheral circulation, and the convenience of liquid biopsy together provide distinct advantages over other common cancer biomarkers [8, 9]. For instance, plasma exosomes bearing CD63, a classic exosome marker [10, 11], and Cav1—which is elevated in prostate cancer and melanoma [12, 13]—are useful melanoma markers and are associated with poor prognosis in melanoma [14]. In pancreatic cancer, GPC1(+) circulating exosomes may serve as a noninvasive tool to enable the early detection of cancer and guide treatment decisions [15]. However, exosomes derived from various body fluids of patients with cancer are a heterogeneous mix of vesicles, as they are likely sourced from both tumor cells and normal cells [16]. Thus, it is essential to identify exosome markers to distinguish between cancer and normal sources. In this way, the underlying mechanisms of TEXs-related modulation of the TME, tumor progression, and metastasis could be unveiled.

Despite recent progress in approaches for TEXs isolation and analysis of corresponding cargos, the roles of TEXs as potential diagnostic or prognostic biomarkers remain unconfirmed. Immune checkpoint blockers (ICBs) have revolutionized tumor treatment strategies by tackling the tumor evasion of the host immune response. Despite the clinical success of ICBs in multiple cancers, favorable and durable response merely occurred in a subset of patients. Considering the practicality and cost-effectiveness, exosome estimation may be a promising diagnostic tool and indicator for ICBs. Gang Chen et al. reported that metastatic melanoma released a high level of exosomal PD-L1 in early stage of treatment associated with immunosuppression, which might stratify potential responders to ICBs therapy [17]. Recently, several clinical trials have investigated whether plasma exosomes can be used as a novel diagnostic and screening indicator in ICBs therapy. For instance, NCT03854032 assessed the dynamic exosomes amounts as well as exosomal PD-L1 levels under the treatment of nivolumab and indoleamine 2, 3-dioxygenase 1 inhibitor in patients with head and neck cancer [18]. Therefore, despite insufficient evidence in the clinical validation, exosomes exhibited great potential as robust biomarkers for predicting response to ICBs.

Single-cell RNA-sequencing analysis is an emerging technique that provides gene expression profiles at the resolution of an individual cell [19], thus enabling identification of various cell types and investigation of key genes and pathways involved in cancer progression and resistance [20, 21]. Considering the lack of studies focusing on exosomes from single-cell platform, we sought to explore exosome-associated mechanisms by utilizing single-cell RNA-seq data.

In current study, we integrated exosomes associated genes including exosome proteins identified in malignant pleural effusion, classical exosome markers, and operators in regulating TEXs secretion. A tumor-derived exosome-related signature, termed TEXscore, was established by utilizing a principal component analysis (PCA) algorithm to predict overall survival outcomes across various cancers. Additionally, the predictive power of TEXscore for ICBs was further investigated. High TEXscore was associated with immunosuppressive microenvironment. Moreover, combining these analyses with noncoding RNA data and genome data, we also revealed TEXscore relevant mechanisms.

## Materials and methods

### Establishment of TEXscore

For selected tumor-derived exosome-associated genes, the expression of each gene was first transformed into a z-score. Then, PCA analysis was conducted, and the principal component 1 was identified as the original score. This approach focuses the score on the set with the largest block of well-correlated (or anti-correlated) genes in the set, while down-weighting contributions from genes that do not track with other set members. The PCA algorithm has been used to establish model signatures in our previous work [22, 23]. In the current study, the PCA algorithm was performed utilizing the IOBR R package [24]. The TEXscore could be calculated reproducibly using the IOBR “calculate_sig_score” function by providing a list of TEXscore genes.

### Single-cell RNA-sequencing data preprocessing

The raw data of single-cell RNA-sequencing from a previous study of Ashley Maynard et al. [20] were downloaded from The National Center for Biotechnology Information (NCBI) BioProject (https://www.ncbi.nlm.nih.gov/bioproject/PRJNA591860). These data included 49 clinical biopsies obtained from 30 patients with metastatic lung cancer before and during targeted therapy. A list of cell types was annotated in the single-cell RNA-sequencing data, a total of 3754 cancer cells were determined by inferCNV. Similar to the TEXscore construction methods described above, TEXscore was calculated for each cancer cell from the clinical biopsies.

### Data source and preprocessing

Data from The Cancer Genome Atlas (TCGA) were downloaded from the UCSC Xena browser (https://gdc.xenahubs.net). To enhance comparability between the samples, RNA-sequencing count data were transformed into Transcripts Per Million (TPM) [25] to calculate gene signature scores. Detailed clinical information for TCGA datasets was obtained via the R package TCGAbiolinks [26]. To further validate survival difference depending on TEXscore, the independent cohorts downloaded from Gene Expression Omnibus (GEO) were analyzed: Cristescu R and colleagues’ gastric cancer data (GSE62254) and Rousseaux S and colleagues’ lung cancer data (GSE30219). The RNA-sequencing data of multistep process of lung squamous carcinogenesis were downloaded under GSE33479. The transcriptome and clinical data of the IMvigor210 dataset from patients with metastatic urothelial cancer treated with an anti-PD-L1 agent (atezolizumab) were downloaded from http://research-pub.gene.com/IMvigor210CoreBiologies. Except from IMvigor210, other cohorts of RNA-seq on patients undergoing ICB included patients with melanoma treated with nivolumab (GSE91061), patients with metastatic melanoma treated with PD-1 inhibitor (GSE78220), and patients with renal cell carcinoma treated with anti-PD-1 (nivolumab) immunotherapy (GSE67501). Due to different platforms and methods of RNA-sequencing, various normalization methods were performed in the GEO datasets. For the GSE33479 and GSE67501 cohorts, normalized data were provided by source reference. For the GSE91061 and GSE78220 cohorts, data were normalized to fragments per kilobase of transcript per million fragments sequenced (FPKM). For the GSE30219 and GSE62254 cohorts, gene expression data were generated from Affymetrix platform, so the data were normalized using robust multiarray averaging (RMA) algorithm. Genomic data were analyzed using R (version 3.5.0) and R Bioconductor packages.

### Inference of infiltrating cell infiltration in the tumor microenvironment

To quantify the proportions of immune cells, we used the Cell type Identification By Estimating Relative Subsets Of RNA Transcripts (CIBERSORT) algorithm [27] which allows for sensitive and specific discrimination of 22 human immune cell phenotypes, including B cells, T cells, natural killer cells, macrophages, dendritic cells, and myeloid subsets. Gene-expression profiles were prepared using standard annotation files, and data were uploaded to the CIBERSORT web portal (http://cibersort.stanford.edu/), with the algorithm run using the 22 human immune cell phenotype signature and 1000 permutations. To characterize other immune microenvironment and prevalent gene signatures activation in each tumor samples, PCA algorithm was applied to determine the pathway activity using gene sets curated by Mariathasanet al. [28], Cristescu et al. [29], Rooney et al. [30], Rosario et al. [31] and Zeng et al. [22]. We thereby obtained, for each signature, an enrichment score per sample that indicated the extent of upregulation or downregulation of the associated genes. A minimum overlap of two genes was required.

### Differentially expressed gene (DEGs) analysis

DEGs were determined using the R package DESeq2 [32]. DEG analysis was conducted using a generalized linear model with the Wald statistical test with R package DESeq2, under the assumption that the underlying gene expression count data were distributed per negative binomial distribution. DEGs satisfying the significance criteria (adjusted *P*-value < 0.05) were selected for further analysis. The Benjamini–Hochberg correction was applied to calculate the adjusted *P* value for multiple tests [33].

### Functional and pathway enrichment analyses

Gene annotation enrichment analysis was conducted using the clusterProfiler package [34]. Gene Ontology (GO) [35] and Kyoto Encyclopedia of Genes and Genomes (KEGG) [36] terms were identified with a strict cutoff of *P* < 0.01 and a false-discovery rate (FDR) of less than 0.05.

### Identification of target genes of TEXscore negative associated miRNAs

The top 10 TEXscore negative associated miRNAs were identified according to the correlation analysis (Additional file 7: Table S2). The downstream target genes list of above miRNAs were downloaded from TargetScan (http://www.targetscan.org/vert_72/) and miRDB [37] (http://www.mirdb.org/). The intersection of both target lists from TargetScan and miRDB were taken for further analyzes.

### Identification of TEXscore relevant mutations

The Mutation Annotation Format (MAF) files were downloaded with TCGAbiolinks, and the mutation status was inferred from the MAF files. Mann–Whitney *U* test was applied to define the significance of binary variables (wild type or mutated). Benjamini–Hochberg method was conducted to convert the *P* values to the adjusted *P* values. The R package maftools was used to generate mutation landscape of TEXscore genes in the TCGA-LUAD and TCGA-LUSC cohorts.

### Statistics

The normality of the variables was tested utilizing the Shapiro–Wilk normality test [38]. For comparisons of two groups, statistical significance for normally distributed variables was estimated via unpaired Student’s t test, and non-normally distributed variables were analyzed via the Mann–Whitney *U* test. For comparisons of more than two groups, Kruskal–Wallis and one-way ANOVA were applied for non-parametric and parametric variables, respectively [39]. The correlation coefficient was calculated using Spearman and distance correlation analyses. Chi-square test and two-sided Fisher’s exact tests were used to analyze contingency tables. Using the R package survminer, the cutoff values of each dataset were computed based on the association between the survival outcome and TEXscore in each independent dataset. The Kaplan–Meier method was used to depict survival curves for the subgroups in each dataset, and the log-rank test was used to determine statistically significant differences. The univariate Cox proportional-hazards regression model was applied to compute the hazard ratios for univariate analyses. The sensitivity and specificity of the signature scores were depicted by the receiver operating characteristic curve (ROC) and quantified by the area under the ROC (AUC) using the R package pROC [40]. All statistical analyses were conducted using R (https://www.r-project.org/), and the *P*-values were two-sided. *P* values lower than 0.05 were considered statistically significant.

## Results

### The construction of tumor-derived exosome associated score

**Fig. 1:**
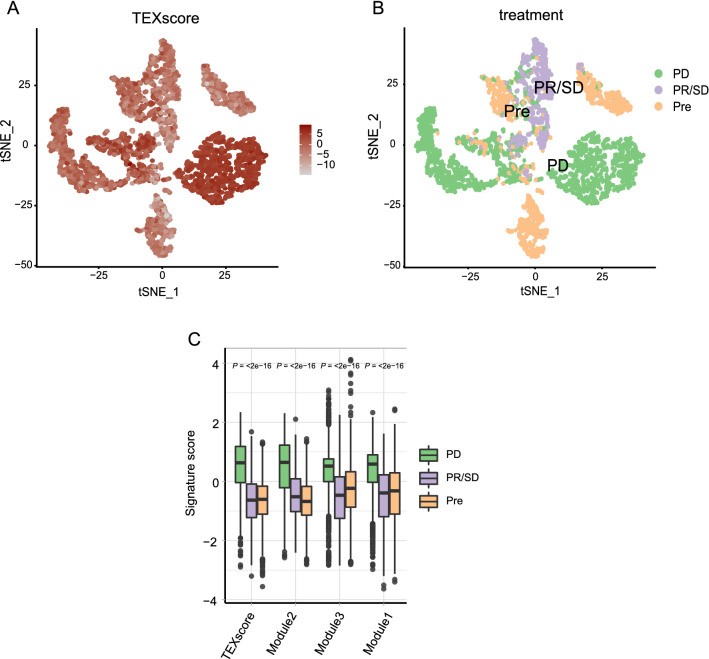
Single-cell transcriptome analysis reveals TEXscore distribution in NSCLC during targeted therapy. **A** t-SNE plots showing expression level of TEXscore in patients with NSCLC. **B** t-SNE plots showing cancer cells in patients with NSCLC colored by treatment time point (PD, PR/SD, and Pre). **C** Exosome-associated signature scores correlated with treatment time point PD during targeted therapy. The statistical difference of three gene clusters was evaluated using Kruskal–Wallis test. P values are indicated

To characterize tumor-derived exosomes, a total of 33 tumor-derived exosome-associated genes were selected and divided into three modules (Additional file 7: Table S1). As reported in a previous study [41], Module 1 contains genes from an extracellular vesicles-associated gene signature of which proteins detected in extracellular vesicles of malignant pleural effusion. Genes in Module 2 included membrane transport and fusion proteins (FLOT1), tetraspanins (CD9, CD63, CD81), chaperones (HSP70), integrins (ITGA1, ITGB1), and multivesicular body synthesis proteins (ALIX, TSG101), which have been widely accepted as classic exosome marker genes in multiple studies [5, 42, 43]. To expand the universality of TEX for other cancers, genes manipulating TEX secretion summarized by McAndrews et al. were added in Module 3 [43]. The GO enrichment analysis and KEGG enrichment analysis of the selected genes revealed that TEX genes were enriched in pathways associated with exosome formation and release (exosomal secretion, extracellular exosome biogenesis, extracellular vesicle biogenesis, focal adhesion, and regulation of actin cytoskeleton). In addition, tumor progression pathway, such as PI3K-Akt signaling pathway, significantly correlated with TEX genes (Additional file 1: Figure S1A, B). Therefore, we considered that the above selected genes were associated with exosome formation and secretion characteristics and tumor progression features. Next, utilizing PCA algorithm to integrate the selected genes, we defined a tumor-derived exosome-associated signature (TEXscore), which could also reflect TEX features to some extent. First, we sought to examine the TEXscore in a single cancer cell dimension. From the single-cell RNA-seq data of patients with non-small cell lung cancer (NSCLC) undergoing tyrosine kinase inhibitor (TKI) therapy, we pooled 3754 cancer cells together, resulting in several clusters via unsupervised graph-based cluster analysis. For each cancer cell, TEXscore was calculated and depicted in Fig. 1A. To delineate the different treatment statuses during therapy, three key time points were stressed. Notably, TEXscores of cancer cells increased at progressive disease (PD) status when tumor acquired drug resistance, compared with the relatively low TEXscores at the time point of pre-treatment (Pre) and partial response (PR)/stable disease (SD) statuses, implying an association between TEXscore and cancer progression undergoing treatment (Kruskal–Wallis test, *P* ≤ 2e−16; Fig. 1B, C). Additionally, exosomes-associated signatures generated from the three modules (Additional file 7: Table S1) separately were established using PCA algorithm, leading to a similar conclusion that TEXscore and exosomes-associated signature scores elevated significantly in PD status (Fig. 1C).

### TEXscore differs in tumor samples and normal tissues

**Fig. 2:**
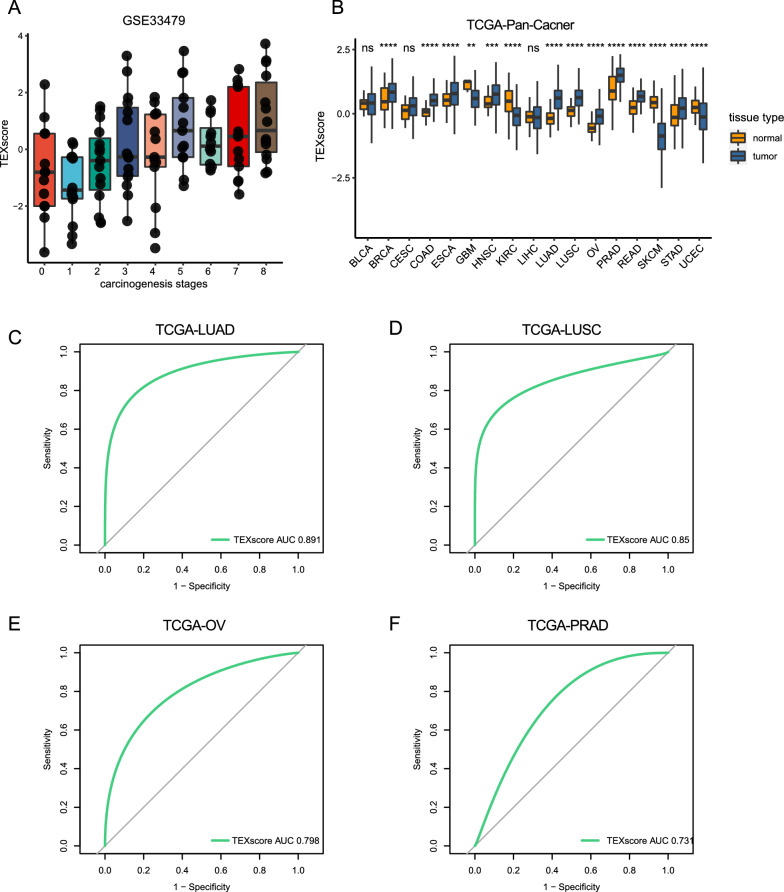
TEXscore holds promise in discriminating of tumor and normal tissues in the TCGA-Pan-Cancer cohort. **A** TEXscore increased along with the ascending malignancy degrees of lung tissues in GSE33479. Spearman rank correlation rho = 0.3968921, *P* = 6.011e−06. **B** TEXscore was elevated in most cancer types in the tumor tissues compared with normal tissues in the TCGA-Pan-Cancer cohort. **C–F** The capacity to identify tumor sources or normal tissues sources of TEXscore in the (**C**) TCGA-LUAD cohort, (**D**) TCGA-LUSC cohort, (**E**) TCGA-OV cohort, and (**F**) TCGA-PRAD cohort. (TCGA-LUAD: AUC = 0.891; TCGA-LUSC: AUC = 0.85; TCGA-OV: AUC = 0.798; TCGA-PRAD: AUC = 0.731.) *P*-values are shown with ****, ***, **, ns representing *P* < 0.0001, *P* < 0.001, *P* < 0.01, no significant, respectively

We sought to assess TEXscore in tumor samples and normal tissues. In the GSE33479 cohort [44], stages 0 and 1 represented normal bronchial mucosa tissues, with increasing degree of malignancy at more advanced stages, where stage 8 subsumed lung squamous carcinoma tissues. Intriguingly, TEXscore maintained a linear increase from normal tissue to invasive tumor samples (Fig. 2A). Next, we tried to validate the different TEXscore levels between tumor samples and normal tissues in a pan-cancer survey. In the TCGA-Pan-Cancer cohort, TEXscore increased in most cancer types—except for TCGA-Glioblastoma (TCGA-GBM), TCGA-Melanoma (TCGA-SKCM), and TCGA-Kidney Clear Cell Carcinoma (TCGA-KIRC)—indicating that TEXscore correlated with tumor characteristics (Fig. 2B). Especially, TEXscore enabled the discrimination of tumor and normal tissues with a considerably higher predictive efficacy in TCGA-Lung Adenocarcinoma (TCGA-LUAD), TCGA-Lung Squamous Cell Carcinoma (TCGA-LUSC), TCGA-Ovarian Cancer (TCGA-OV), and TCGA-Prostate Cancer (TCGA-PRAD) cohorts (TCGA-LUAD: AUC = 0.891, TCGA-LUSC: AUC = 0.85, TCGA-OV: AUC = 0.798, TCGA-PRAD: AUC = 0.731; Fig. 2C–F).

### Pan-cancer survey of TEXscore and associations with prognosis

**Fig. 3:**
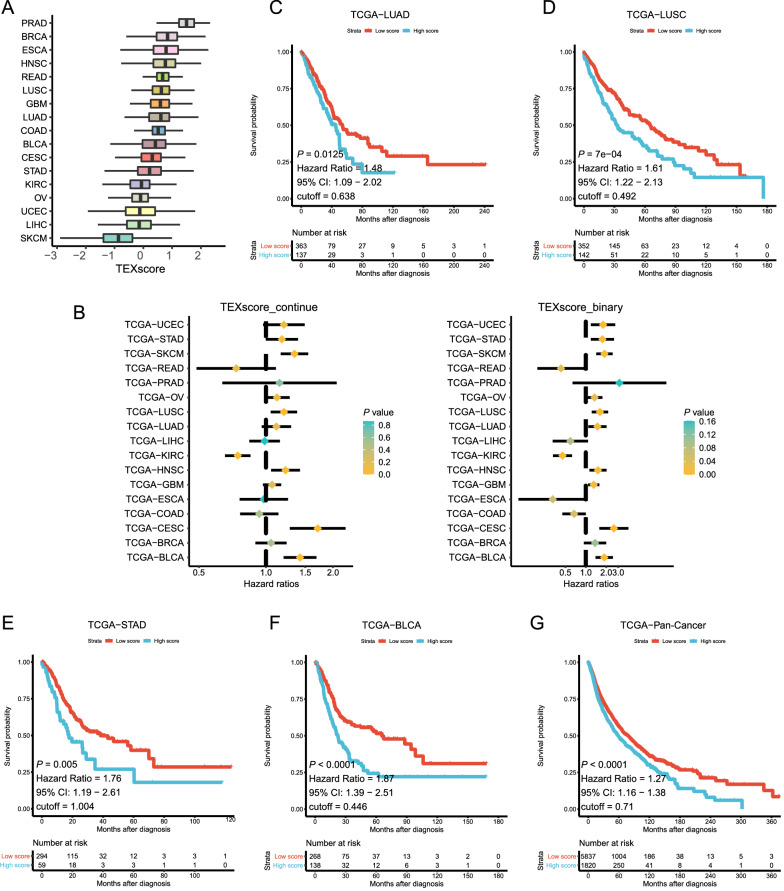
Pan-cancer survey of TEXscore and associations with survival in TCGA. **A** Boxplots show individual values of TEXscore in each cancer type ranked by median TEXscore. **B** The forest plot exhibits the hazard ratios of TEXscore in TCGA-Pan-Cancer. The TEXscore served as an unfavorable factor (hazard ratio > 0) for the prognosis of most cancer types in the TCGA datasets. **C** The Kaplan–Meier survival analysis indicated a poor overall survival of high-TEXscore patients (high: blue; low: red) in the TCGA-LUAD dataset (*P* = 0.0125, Hazard ratio = 1.48, 95% CI = 1.09–2.02). **D** The Kaplan–Meier survival analysis indicated a poor overall survival of high-TEXscore patients (high: blue; low: red) in the TCGA-LUSC dataset (*P* = 7e-04, Hazard ratio = 1.61, 95% CI = 1.22–2.13). **E** The Kaplan–Meier survival analysis indicated a poor overall survival of high-TEXscore patients (high: blue; low: red) in the TCGA-STAD dataset (*P* = 0.005, Hazard ratio = 1.76, 95% CI = 1.19–2.61). **F** The Kaplan–Meier survival analysis indicated a poor overall survival of high-TEXscore patients (high: blue; low: red) in the TCGA-BLCA dataset (*P* < 0.0001, Hazard ratio = 1.87, 95% CI = 1.39–2.51). **G** The Kaplan–Meier survival analysis indicated a poor overall survival of high-TEXscore patients (high: blue; low: red) in the TCGA-Pan-Cancer dataset (*P* < 0.0001, Hazard ratio = 1.27, 95% CI = 1.16–1.38)

Exosomes are considered attractive noninvasive biomarkers across cancer types, especially for those with specific tumor biomarkers detected in various body fluids. Here, we showed the pan-cancer TEXscore in 17 TCGA cancer types, with cancer types ranked by a median TEXscore (Fig. 3A). High TEXscores were displayed in TCGA-PRAD, TCGA-Breast Cancer (TCGA-BRCA), TCGA-Esophageal Cancer (TCGA-ESCA), TCGA-Head and Neck Cancer (TCGA-HNSC), TCGA-Rectal Cancer (TCGA-READ), TCGA-LUSC, TCGA-GBM, and TCGA-LUAD, suggesting that these tumors were prone to generating more TEX (Fig. 3A). As expected, high TEXscore (both as a continuous variable and as a categorical variable) was associated with worse clinical outcomes, indicating its great capacity for predicting overall survival in most cancer types, especially for TCGA-LUSC, TCGA-LUAD, TCGA-Stomach Cancer (TCGA-STAD), and TCGA-Bladder Cancer (TCGA-BLCA) (TCGA-LUAD: *P* = 0.0125, Hazard Ratio = 1.48, 95% CI: 1.09−2.02; TCGA-LUSC: *P* = 7e−04, Hazard Ratio = 1.61, 95% CI: 1.22–2.13; TCGA-STAD: *P* = 0.005, Hazard Ratio = 1.76, 95% CI: 1.19−2.61; TCGA-BLCA: *P* < 0.0001, Hazard Ratio = 1.87, 95% CI: 1.39−2.51; Fig. 3B–F; Additional file 2: Figure S2A–H). Furthermore, similar trend for TEXscore was validated in gastric cancer (GSE62254) and lung cancer (GSE30219) through the Kaplan–Meier survival analysis (Additional file 2: Figure S2I, J). Although TCGA-Colon Cancer (TCGA-COAD), TCGA-ESCA, TCGA-Liver Cancer (TCGA-LIHC), TCGA-KIRC, and TCGA-READ revealed inverse survival differences, only the latter two types showed statistically significant differences (Additional file 3: Figure S3). In the integrated cohort of TCGA pan-cancer, TEXscore illustrated the robust predictive value for overall survival (TCGA-Pan-Cancer: *P* < 0.0001, Hazard Ratio = 1.27, 95% CI: 1.16–1.38; Fig. 3G).

Multi-variate Cox regression also recognized TEXscore as a prognostic factor in TCGA-LUSC, TCGA-STAD, and TCGA-BLCA cohort (Additional file 7: Table S3). Given the statistical survival difference in TCGA-LUSC, TCGA-LUAD, TCGA-STAD, and TCGA-BLCA, these four cancer types were selected for consequent analysis.

### TEXscore predicts therapeutic response to immune checkpoint blocker

**Fig. 4:**
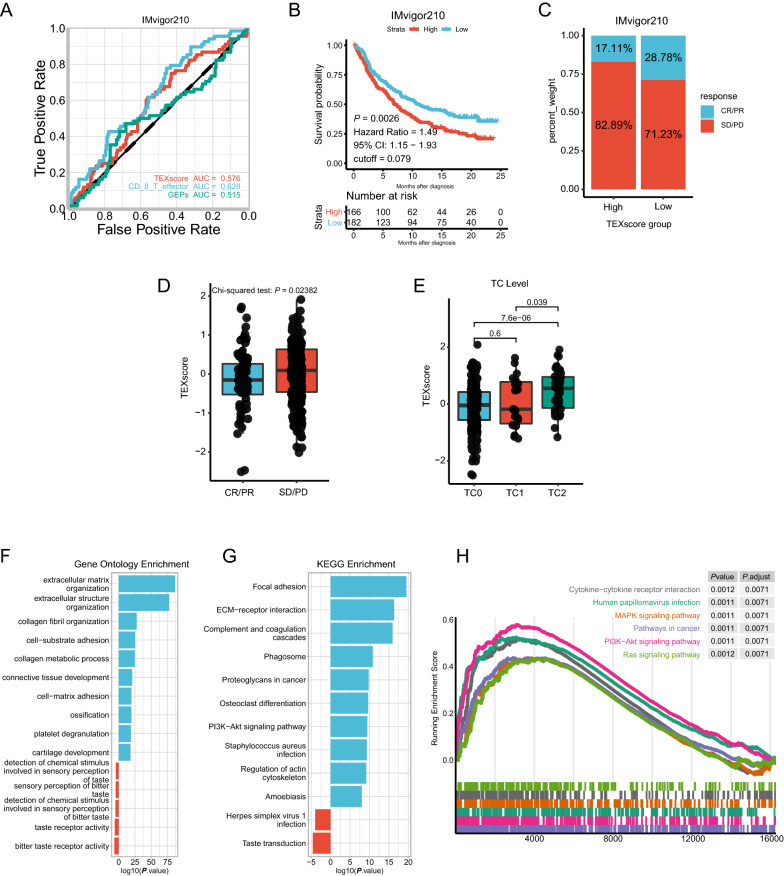
TEXscore predicts benefit from immune checkpoint blockers therapy. **A** ROC analyses suggested the TEXscore exerted inferior predictive sensitivity to anti-PD-L1 response compared with CD8 + T cells and GEP in IMvigor210 dataset. (AUC = 0.576, 0.628, 0.515, respectively). **B** The Kaplan–Meier survival analysis indicated a poor overall survival of high-TEXscore patients (high: red; low: blue) in the IMvigor210 dataset (*P* = 0.0026, Hazard ratio = 1.49, 95% CI = 1.15–1.93). **C** Rate of clinical response (complete response [CR]/partial response [PR] and stable disease [SD]/progressive disease [PD]) to anti–PD-L1 immunotherapy in high or low TEXscore groups in the IMvigor210 dataset (chi-square test, *P* = 0.02382). **D** Distribution of TEXscore in different clinical responses (CR/PR, SD/PD) in the IMvigor210 dataset (Chi-square test, *P* = 0.02382). **E** PD-L1 expression (TC level) were associated with TEXscore. *P* values are indicated. Tumor cells (TC) level was measured to evaluate PD-L1 expression on tumor cells. The specimens were scored as IHC TC0, TC1, or TC2 if < 1%, ≥ 1% but < 5%, or ≥ 5% of TC were PD-L1 positive, respectively. **F** GO enrichment analysis of the DEGs between high (blue) and low (red) TEXscore in IMvigor210 dataset. The x-axis indicates the log10 (*P* value). **G** KEGG enrichment analysis of the DEGs between high (blue) and low (red) TEXscore in the IMvigor210 dataset. The x-axis indicates the log10 (*P* value). **H** GSEA analyses displayed key pathways enriched in high (up) TEXscore group. Gene sets that are inferred to reflect key underlying biological processes are colored

Given the remarkable success of ICB therapy, numerous predictive biomarkers have been identified to distinguish candidate patients who can benefit from ICB, but their predictive capacities are limited for diverse reasons. Herein, we sought to explore the predictive value of TEXscore for ICB. TEXscore presented non-inferior predictive capacity for ICB in melanoma (GSE91061 and GSE78220) compared with CD8 + T effector and T cell inflamed gene expression profile (GEP), which commonly associated with immune activation (Additional file 4: Figure S4A–D). Furthermore, predictive capacity of TEXscore exceeded that of the CD8 + T effector, and T cell-inflamed GEP in anti-PD-1 (nivolumab) immunotherapy in renal cell carcinoma (GSE67501) (Additional file 4: Figure S4E–F). Since TEXscore exhibited promising power in predicting prognosis in bladder cancer, IMvigor210 dataset of patients with metastatic urothelial cancer undergoing anti-PD-L1 therapy was used to analyze the performance of TEXscore in screening patients with potential therapeutic benefit. Despite the lack of a satisfying AUC for TEXscore, the Kaplan–Meier curve demonstrated that a higher TEXscore was linked to prolonged survival time in IMvigor210 (TEXscore: AUC = 0.576; CD8 + T effector: AUC = 0.628; GEPs: AUC = 0.515; IMvigor210: *P* = 0.0026, Hazard Ratio = 1.49, 95% CI: 1.15–1.93; Fig. 4A, B). Consistently, TEXscore was elevated in nonresponders (SD/PD) to ICB (chi-square test, *P* = 0.02382; Fig. 4C, D). Notably, TEXscore increased as the tumor cell (TC) PD-L1 level, reflecting PD-L1 expression on the tumor cells, elevated instead of immune cell (IC) PD-L1 level, thus further verifying that TEXscore was a tumor-specific biomarker (Fig. 4E; Additional file 4: Figure S4G). To identify more underlying mechanisms contributing to its predictive power for ICB, DEGs between high- and low-TEXscore subgroups in the IMvigor210 cohort were used to perform functional enrichment analysis. The Gene Ontology enrichment and KEGG enrichment analyses unanimously validated that the genes upregulated in the high-TEXscore subgroup were enriched in extracellular matrix (ECM) remodeling-associated pathways, suggesting that the alterations in ECM components could be triggered by exosome release and uptake process [45] (Fig. 4F, G). Moreover, Gene set enrichment analysis (GSEA) results and KEGG analysis together clarified that genes upregulated in the high-TEXscore subset were involved in tumor-related signaling pathways, including PI3K-Akt signaling pathway, MAPK signaling pathway, and pathway in cancer (Fig. 4F–H). These pathways emphasized the malignant features of tumor that were responsible for the unsatisfactory response during the ICB treatment.

### TEXscore correlates with immunosuppressive microenvironment

Cancer cells deliver immune signaling molecules via exosomes to modify the tumor microenvironment [46]. The abovementioned results indicated that TEXscore correlated with poor clinical outcomes in patients undergoing ICB, which implied an underlying association between TEXscore and immunosuppressive microenvironment. To gain insight into the exact effects that exosomes exert in the tumor microenvironment, we first conducted CIBERSORT to investigate the immune cell infiltration pattern mediated by exosomes. Intriguingly, higher TEXscores were accompanied with rising M0 and M2 macrophage infiltration in TCGA-LUSC, TCGA-LUAD, TCGA-STAD, and TCGA-BLCA, albeit with bare statistical significance; in contrast, CD8 + T cells level negatively correlated with TEXscore, implying that TEX contributed to remodeling of the immunosuppressive microenvironment (Additional file 5: Figure S5A–D).

**Fig. 5:**
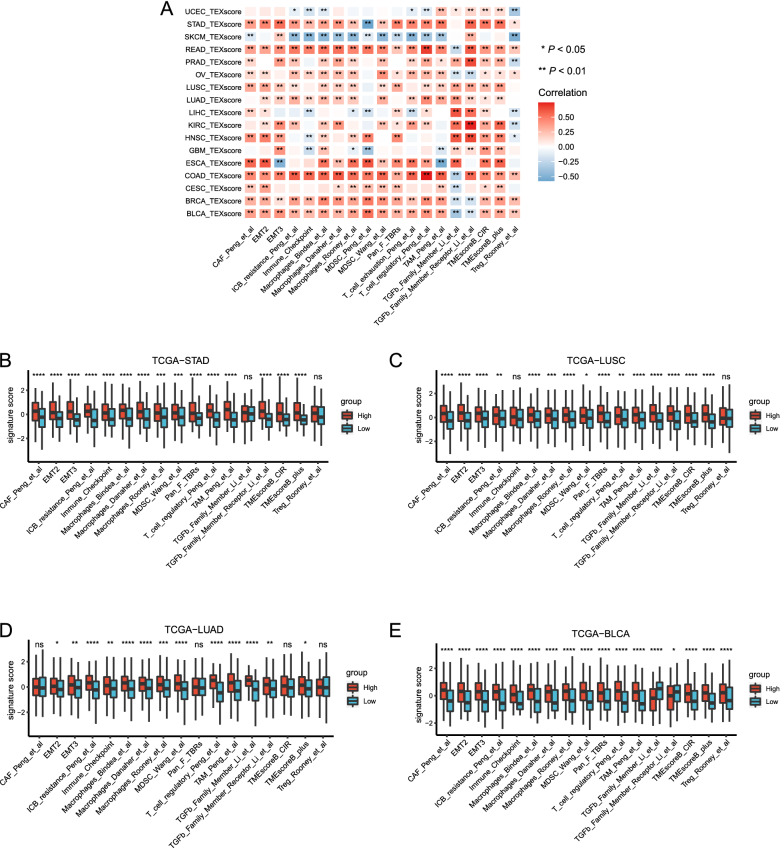
TEXscore correlates with immunosuppressive microenvironment. **A** A corplot exhibited the correlations between the TEXscore and immunosuppressive signature scores in the TCGA-Pan-Cancer cohort. The coefficient was continuous and was represented using colors from blue (negative) to red (positive). *P*-values are shown with ****, ***, **, * representing *P* < 0.0001, *P* < 0.001, *P* < 0.01, *P* < 0.05, respectively. **B–E** A high (red) TEXscore was associated with an increase in the immunosuppressive signature score compared with that in the low (blue) TEXscore group in (**B**) TCGA-STAD, (**C**) TCGA-LUSC, (**D**) TCGA-LUAD, and (**E**) TCGA-BLCA datasets, respectively

Subsequently, we integrated comprehensive immunosuppressive signatures covering macrophages, cancer-associated fibroblasts (CAFs), myeloid-derived suppressor cells (MDSCs), epithelial–mesenchymal transformation (EMT), and regulatory T cells (Tregs) to characterize the relationship between TEXscore and the tumor microenvironment. The immunosuppressive microenvironment pattern was significantly distinct across different tumor types in TCGA datasets (Fig. 5A). Namely, TCGA-SKCM, TCGA-Endometrioid Cancer (TCGA-UCEC), TCGA-LIHC, and TCGA-GBM showed a negative correlation with the immunosuppressive signatures compared with most cancer types, so there might be considerable intertumor heterogeneity in terms of TEX secretion status. Meanwhile, TEXscore was markedly related to the extensive immunosuppressive signatures in most cancer types as well as to immune therapy resistance signatures, such as immune resistance signature developed by Peng et al. [47], and TMEscoreB [22], which had been confirmed to be nonsensitive markers of ICB (Fig. 5B–E). Additionally, immunosuppressive cells including CAFs, MDSCs, and macrophages positively correlated with TEXscore in the IMvigor210 dataset, collectively validating that TEXscore served as a negative indicator for ICB (Additional file 6: Figure S6A–D).

### TEXscore-associated miRNAs mediate tumor biological mechanisms

**Fig. 6:**
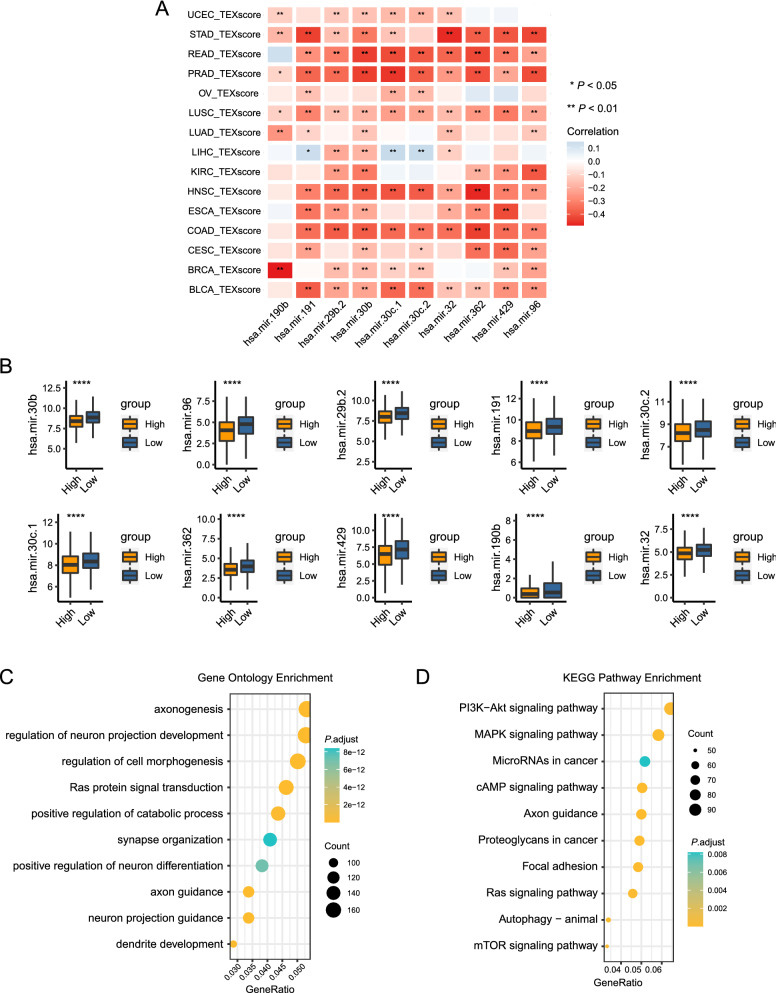
TEXscore-associated miRNAs mediate tumor biological mechanisms. **A** A corplot displayed the correlations between the TEXscore and top 10 negatively associated miRNAs levels in the TCGA-Pan-Cancer cohort. The coefficient was continuous and was represented using colors from red (negative) to blue (positive). *P*-values are shown with **, * representing *P* < 0.01, *P* < 0.05, respectively. **B** Top 10 negatively associated miRNAs levels decreased in the high-TEXscore settings. *P*-values are shown with **** representing *P* < 0.0001. **C** GO enrichment analysis of the target genes of top 10 negatively associated miRNAs. Key GO terms are shown ranked by counts. **D** KEGG enrichment analysis of the target genes of top 10 negatively associated miRNAs. Key KEGG pathways are shown ranked by counts

Aberrant miRNA activities are common in diverse cancer settings. Currently, massive studies focused on the function of miRNAs as exosomal cargos. Therefore, we sought to investigate the relationship between TEXscore and miRNAs. A total of 1882 miRNAs in the TCGA-Pan-Cancer cohort were included to dissect their relationship with TEXscore. The top 10 miRNAs with negative correlation are summarized in Additional file 7: Table S2. Digestive system tumors and urinary system tumors, such as TCGA-STAD, TCGA-READ, TCGA-COAD, TCGA-BLCA, and TCGA-PRAD, exhibited wide links with those miRNAs (Fig. 6A). Notably, these identified miRNAs mainly functioned as tumor suppressors, and they were downregulated in the high-TEXscore group (Fig. 6B). Since miRNAs can repress specific genes expression by binding to the 3′ untranslated regions (3'UTRs) of target mRNAs, we sought to obtain the target mRNA lists of the above miRNAs. To further dissect the functions exerted by TEXscore-associated miRNAs in the high-TEXscore settings, a total of 2628 target genes of top 10 negative miRNAs were selected for further GO and KEGG enrichment analyses. Intriguingly, GO enrichment analysis demonstrated that the target mRNAs were enriched in neurogenesis processes, including axonogenesis, synapse organization, and neuron projection (Fig. 6C). Apart from ECM remodeling mechanism, KEGG enrichment analysis provided new insight that decreased miRNAs in the high-TEXscore group participated in various tumorigenesis signaling pathways (Ras signaling pathway, PI3K-Akt signaling pathway, and MAPK signaling pathway) (Fig. 6D).

### TEXscore is related to stable genomic condition

**Fig. 7:**
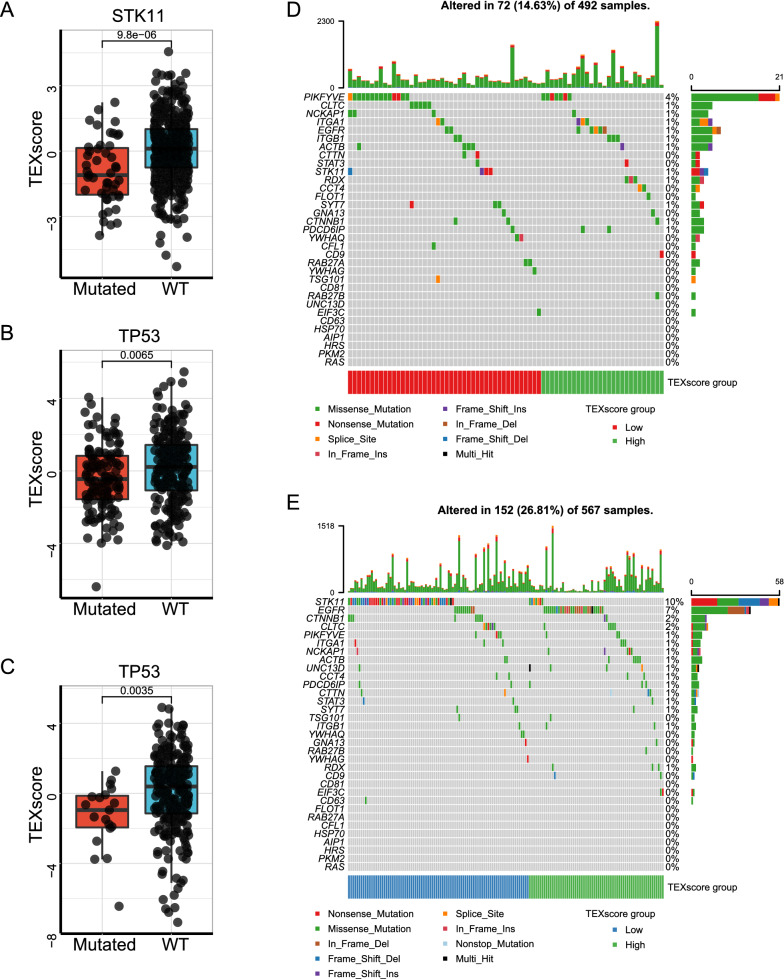
TEXscore is related to stable genomic condition. **A** Wild-type (WT) STK11 was significantly associated with higher TEXscore compared with mutation status in the TCGA-LUAD cohort (Mann–Whitney *U* test, *P* = 9.8e−06). **B** WT TP53 was significantly associated with higher TEXscore compared with mutation status in the TCGA-STAD cohort (Mann–Whitney *U* test, *P* = 0.0065). **C** WT TP53 was significantly associated with higher TEXscore compared with mutation status in the TCGA-CESC cohort (Mann–Whitney *U* test, *P* = 0.0035). **D**, **E** The oncoPrint showing the mutation status of TEXscore genes was constructed by those with low TEXscore on the left and those with high TEXscore on the right in the (**D**) TCGA-LUSC cohort and (**E**) TCGA-LUAD cohort

We analyzed the genomic profile of TEXscore and common genes. TEXscore significantly decreased in STK11 mutation settings in the TCGA-LUAD cohort (Mann–Whitney *U* test, *P* = 9.8e−06; Fig. 7A), consistent with prior research that wild-type LKB1 restoration enhanced exosomes release [48]. Similarly, wild-type TP53, which played a vital role in exosome biogenesis, was tightly associated with TEXscore in the TCGA-STAD and TCGA-Cervical Cancer (TCGA-CESC) cohorts (Mann–Whitney *U* test, *P* = 0.0065, *P* = 0.0035, respectively; Fig. 7B, C). Next, we concentrated on the whole TEXscore genes alteration rate. The genes from TEXscore kept a relatively low mutation rate in the TCGA-BLCA and TCGA-STAD, while NSCLC possessed a higher mutation rate. EGFR mutation is the most frequently encountered driver mutation in NSCLC, especially in lung adenocarcinoma [49]. Remarkably, high EGFR alteration frequency was observed in the high-TEXscore group in the TCGA-LUAD, STK11 mutations predominated in the low-TEXscore group (Fig. 7D–E), which collectively indicated that STK11 and EGFR mediated converse effects on exosome secretion.

## Discussion

Increasing evidence recognizes exosomes as vital regulators in cancer initiation and progression steps; thus, identifying and tracking TEX may facilitate the discovery of exosome-mediated protumorigenic mechanisms in the complex communication system in the tumor microenvironment. Here, a tumor-specific exosome-associated signature named TEXscore was constructed using the PCA algorithm. Comprehensive exploration of massive data derived from TCGA-Pan-Cancer cohorts, IMvigor210 cohort, and six GEO datasets further expanded our understanding of TEX-related tumor-intrinsic features and how TEXs modify the tumor microenvironment.

Exosomes can be detected in multiple human body fluids, such as blood plasma, urine, saliva, and pleural fluid; however, they are usually mixtures of vesicles produced by normal tissues and tumor cells. Despite methods for TEX isolation, such as size-exclusion chromatography and a series of ultracentrifugations [50, 51], it is still difficult to separate TEXs from larger extracellular vesicles. Regarding tumor-specific characteristics, TEXscore was elevated in the tumor tissues compared with normal tissues; moreover, it increased directly with the ascending degree of malignancy of tissue. Furthermore, DEGs, GSEA, and KEGG analyses illustrated that tumors with high TEXscore displayed higher activation of carcinogenesis pathways, including PI3K-Akt and MAPK signaling pathways—which had previously been reported to induce tumor progression, proliferation, and drug resistance [4, 52, 53]. The prognostic value of TEXscore was validated in the different independent cohorts.

The advantage of single-cell analysis derives from its high resolution. Here, we introduced single-cell RNA-seq data and calculated TEXscore of each cancer cell from patients with advanced-stage NSCLC. Our results revealed complex heterogeneity of TEX secretion level among tumor tissues. Intriguingly, TEXscore elevated at the progressive disease status compared with the time point before treatment, implying that TEXscore may function as an indicator of TKI responses in NSCLC. As noted by a prior study [20], the tumor microenvironment exhibited different immune cell infiltration patterns across three time points. The TME at PD was characterized by the immunosuppressive milieu where macrophages and regulatory T cells dominated; this was consistent with immune infiltration in the high-TEXscore setting, suggesting that TEXscore changed with the evolving TME response to TKI treatment.

TME consists of cancer cells, immune cells (i.e., T cells, B cells, macrophages, dendritic cells, and natural killer cells), and non-immune host cells such as fibroblasts, which permits frequent intercellular interaction between tumor and surrounding stromal cells [54–56]. By promoting the immune cells differentiation into immunosuppressive subtype, TEX reprogram resident milieu into to tumor-favoring microenvironment [57, 58]. For example, bladder cancer exosomes-mediated TGF-β triggered the activation of normal fibroblasts into CAFs, thereby supporting the tumor progression [59]. Likewise, TEX also induced CD8 + T cells suppressor phenotype to impair antitumor abilities [6]. Consistent with previous studies, our pan-cancer analysis revealed the elevated levels of CAFs, M2 macrophages, and MDSCs in the high-TEXscore subgroup, supporting the tight correlation between TEXscore and immune inhibitory factors. Given that immunosuppressive molecules can be transferred with exosomes, accumulated exosomes might amplify the immune suppression condition in a cascading way.

To date, the development of predictive biomarkers for ICBs is a rapidly emerging field, but few biomarkers were identified from exosomes. Our findings implied that TEXscore could serve as a potential biomarker to identify patients likely to benefit from ICBs. The Kaplan–Meier analysis verified that patients with a higher TEXscore exhibited worse overall survival in the IMvigor210 cohort. Moreover, TEXscore was not inferior to GEP [29] in predicting response to anti-PD-L1 therapy in the IMvigor210 cohort. Moreover, TEXscore achieved encouraging prediction accuracy in melanoma (GSE78220, GSE91061), non-small cell lung cancer (GSE91061), and renal cell carcinoma (GSE67501), though with bare statistical difference due to the small sample size. Currently, PD-L1 expression remains the most widely accepted criterion for clinical implementation; we showed that PD-L1 was upregulated in the high-TEXscore setting. However, it seems controversial that high PD-L1 expression usually indicates better ICB efficacy, while TEXscore serves as a negative indicator for ICB. Tumor PD-L1 expression originates from both intrinsic and external sources, driven by oncogenic activation and induced by IFNγ production from T cells [60]. PD-L1 subgroup analysis revealed that no significant object response rate was observed when grouped by TC-level in IMvigor210 [61], indicating that PD-L1 might increase initially by oncogenic activation and lead to escape from the antitumor immune response. Recently, Azuma et al. evaluated PD-L1 expression level by immunohistochemistry in surgically resected NSCLC samples; they confirmed that PD-L1 overexpression correlated with activating EGFR mutations [62]. The KEYNOTE-001 study showed that in patients with PD-L1 overexpression (i.e., tumor proportional score [TPS] > 50%), pembrolizumab was less effective in EGFR-mutant tumors than in EGFR wild-type tumors (median overall survival = 6.5 vs. 15.7 months, respectively) [63, 64]. Therefore, PD-L1 elevation in the high-TEXscore condition may be driven by oncogene alterations such as those in EGFR, which are nonsensitive to ICB therapy, also associated with immunosuppressive microenvironment.

Notably, our data identified the top 10 miRNAs negatively correlated with TEXscore. Interestingly, these miRNAs mainly had tumor suppressor roles. Namely, miR-30 family accounting for the majority of the miRNAs has been shown to inhibit tumorigenesis in NSCLC [65], and its overexpression prevents breast cancer bone metastasis [66]. Yet, no studies have reported the function of miR-30 family in terms of exosome in cancer; therefore, our results might suggest a novel mechanism for miRNAs in regulating exosomes formation. miRNAs are short, noncoding RNAs that repress target genes expression via binding the 3′UTRs of mRNAs. Thus, in-depth knowledge of the target genes of the miRNAs would facilitate the understanding of the relationship between these miRNAs and exosomes. Intriguingly, the fact that target genes were relatively enriched in GO terms regarding the innervation process (including axonogenesis, neuron projection, and synapse organization) further proposes that miRNAs might strengthen the correlation between TEXs and innervation to some extent. In line with our findings, Marianna Madeo et al. provided both in vivo and in vitro evidence that tumor-released exosomes potentiated cancer innervation [67], but the reverse mechanism, i.e., whether innervation promoted exosomes generation, requires further validation.

Several limitations in the current study should be acknowledged. First, TEXscore was established based on RNA-seq data from tumor tissues, and it should be optimized for applying in liquid biopsy. Furthermore, our research found that TEXscore was associated with poor prognosis, and TEXscore elevated in tumor samples compared with normal tissues. In the current study, TEXscore signature was established relying on exosome-associated genes integration, thus experimental studies are required to further validate TEXscore genes expression level in exosomes and confirm the relationship between TEXscore and clinical outcomes.

## Conclusions

Overall, our study developed a gene-expression signature (TEXscore) capturing TEX characteristics. Furthermore, TEXscore was remarkably associated with immunosuppressive microenvironment, thereby possessing potential in assessing prognosis and predicting ICB response.

## Supplementary Information

**Additional file 1: Figure S1.** TEXscore is associated with exosome features. (A) The top ten signaling pathways enriched of TEXscore genes by Gene ontology (GO) analysis were shown. (**B**) The top ten signaling pathways enriched of TEXscore genes by KEGG analysis were shown.

**Additional file 2: Figure S2.** High TEXscore correlated with poor overall survival. (A-J) Kaplan‐Meier survival curves suggested that a poor overall survival of patients with high TEXscore (high: blue; low: red) in (**A**) TCGA-BRCA dataset (*P* = 0.105, Hazard ratio = 1.37, 95%CI = 0.94–2), (**B**) TCGA-CESC dataset (*P* = 2e-04, Hazard ratio = 2.59, 95%CI = 1.58–4.24), (**C**) TCGA-GBM dataset (*P* = 0.0056, Hazard ratio = 1.31, 95%CI = 1.08–1.58), (**D**) TCGA-HNSC dataset (*P* = 0.0053, Hazard ratio = 1.51, 95%CI = 1.13–2.02), (**E**) TCGA-OV dataset (*P* = 0.0292, Hazard ratio = 1.35, 95%CI = 1.03–1.76), (**F**) TCGA-PRAD dataset (*P* = 0.1602, Hazard ratio = 3.12, 95%CI = 0.64–15.31), (**G**) TCGA-SKCM dataset (*P* < 0.0001, Hazard ratio = 1.88, 95%CI = 1.43–2.49), (**H**) TCGA-UCEC dataset (*P* = 0.0057, Hazard ratio = 1.79, 95%CI = 1.19–2.72), (**I**) GSE62254 dataset (*P* = 1.08e-06, Hazard ratio = 2.17, 95%CI = 1.54–3.03), (**J**) GSE30219 dataset (*P* = 0.00858, Hazard ratio = 1.45, 95%CI = 1.09–1.96).

**Additional file 3: Figure S3.** High TEXscore correlated with favorable overall survival. (A-E) Kaplan‐Meier survival curves suggested that a better overall survival of patients with high TEXscore (high: blue; low: red) in (**A**) TCGA-COAD dataset (*P* = 0.0512, Hazard ratio = 0.67, 95%CI = 0.45–1), (**B**) TCGA-ESCA dataset (*P* = 0.0584, Hazard ratio = 0.33, 95%CI = 0.1–1.04), (**C**) TCGA-READ dataset (*P* = 0.04, Hazard ratio = 0.43, 95%CI = 0.19–0.96), (**D**) TCGA-KIRC dataset (*P* < 0.0001, Hazard ratio = 0.45, 95%CI = 0.33–0.63), (**E**) TCGA-LIHC dataset (*P* = 0.0874, Hazard ratio = 0.59, 95%CI = 0.33–1.08).

**Additional file 4: Figure S4.** Predictive value of TEXscore towards immune checkpoint blockers in independent cohorts. (**A**) ROC curve suggested that TEXscore exerted inferior predictive capacity to ICB response in GSE91061 cohort. (TEXscore: AUC = 0.64, CD8+ T cells: AUC = 0.622, GEP: AUC = 0.607). (**B**) Rate of clinical response (non-response (NR) and response (R)) to ICB in high or low TEXscore groups in the GSE91061 cohort. (**C**) ROC curve suggested that TEXscore exerted inferior predictive capacity to ICB response in GSE78220 cohort. (TEXscore: AUC = 0.607, CD8 + T cells: AUC = 0.542, GEP: AUC = 0.571). (**D**) Rate of clinical response (NR and R) to ICB in high or low TEXscore groups in the GSE78220 cohort. (**E**) ROC curve suggested that TEXscore exerted inferior predictive capacity to ICB response in GSE67501 cohort. (TEXscore: AUC = 0.75, CD8 + T cells: AUC = 0.607, GEP: AUC = 0.464). (**F**) Rate of clinical response (NR and R) to ICB in high or low TEXscore groups in the GSE67501 cohort. (**G**) IC level represented PD-L1 expression on tumour-infiltrating immune cells through immunohistochemistry (IHC). IC level was not related to the TEXscore. Specimens were scored as IHC IC0, IC1, or IC2 if < 1%, ≥ 1% but < 5%, or ≥ 5% of IC were PD-L1 positive, respectively.

**Additional file 5: Figure S5.** Immune cell infiltration level in high and low TEXscore settings validated by CIBERSORT in TCGA. (**A-D**) High TEXscore was accompanied with alteration of M0 and M2 macrophage infiltration in (**A**) TCGA-BLCA cohort, (**B**) TCGA-LUAD cohort, (**C**) TCGA-LUSC cohort, (D) TCGA-STAD cohort.

**Additional file 6: Figure S6.** Tumor microenvironment signatures in IMvigor210 dataset. (**A**) Expression of CAFs associated signatures elevated in high (red) TEXscore versus the low (blue) in IMvigor210. (**B**) Expression of MDSC associated signatures elevated in high (red) TEXscore versus the low (blue) in IMvigor210. (**C**) Expression of CD4 + T cell associated signatures elevated in low (blue) TEXscore versus the high (red) in IMvigor210. (**D**) Expression of macrophage associated signatures elevated in high (red) TEXscore versus the low (blue) in IMvigor210. *P*-values are shown with ****, ***, **, *, ns representing *P* < 0.0001, *P* < 0.001, *P* < 0.01, *P* < 0.05,  no significant, respectively.

**Additional file 7: Table S1.** Tumor-derived exosomes associated genes. **Table S2.** The top 10 miRNAs inversely correlated with TEXscore. **Table S3.** Multi-variate Cox regression results of TEXscore in TCGA-LUSC, TCGA-STAD, and TCGA-BLCA cohort. **Table S4.** Statistical results of Fig. 5B–E. **Table S5.** Statistical results of Figure S5. **Table S6.** Statistical results of Figure S6.

## Data Availability

The datasets of this article were generated from the TCGA database, GEO database, and IMvigor210 dataset.

## Abbreviations

TEXs: Tumor-derived exosomes
PCA: Principal component analysis
ICB: Immune checkpoint blocker
GEO: Gene expression omnibus
TPS: Tumor proportional score
TPM: Transcripts per million
ECM: Extracellular matrix
CIBERSORT: Cell type identification by estimating relative subsets of RNA transcripts
NCBI: The National Center for Biotechnology Information
GO: Gene ontology
TCGA: The cancer genome atlas
TCGA-BLCA: The Cancer Genome Atlas Bladder Cancer
TCGA-CESC: The Cancer Genome Atlas Cervical Cancer
TCGA-COAD: The Cancer Genome Atlas Colon Cancer
TCGA-UCEC: The Cancer Genome Atlas Endometrioid Cancer
TCGA-ESCA: The Cancer Genome Atlas Esophageal Cancer
TCGA-GBM: The Cancer Genome Atlas Glioblastoma
TCGA-HNSC: The Cancer Genome Atlas Head and Neck Cancer
TCGA-KIRC: The Cancer Genome Atlas Kidney Clear Cell Carcinoma
TCGA-LIHC: The Cancer Genome Atlas Liver Cancer
TCGA-LUAD: The Cancer Genome Atlas Lung Adenocarcinoma
TCGA-LUSC: The Cancer Genome Atlas Lung Squamous Cell Carcinoma
TCGA-OV: The Cancer Genome Atlas Ovarian Cancer
TCGA-PRAD: The Cancer Genome Atlas Prostate Cancer
TCGA-READ: The Cancer Genome Atlas Rectal Cancer
TCGA-STAD: The Cancer Genome Atlas Stomach Cancer
TCGA-SKCM: The Cancer Genome Atlas Melanoma
TCGA-BRCA: The Cancer Genome Atlas Breast Cancer
TME: Tumor microenvironment
RMA: Robust multiarray averaging
KEGG: Kyoto Encyclopedia of Genes and Genomes
FDR: False discovery rate
NSCLC: Non-small cell lung cancer
TKI: Tyrosine kinase inhibitor
GEP: Gene expression profile score
ROC: Receiver operating characteristic curve
AUC: Area under curve
GSEA: Gene set enrichment analysis
PD-L1: Programmed cell death ligands 1
PD-1: Programmed cell death protein 1
MDSC: Myeloid-derived suppressor cells
CAFs: Cancer-associated fibroblasts
MAF: Mutation Annotation Format
3′UTRs: 3′ Untranslated regions
EMT: Epithelial–mesenchymal transformation
