# Supplementary figures and images for: Single-cell analysis of a tumor-derived exosome signature correlates with prognosis and immunotherapy response
Source: J Transl Med. 2021 Sep 8;19:381. doi: 10.1186/s12967-021-03053-4 (PMC8424990; doi:10.1186/s12967-021-03053-4)

A

## Gene Ontology Enrichment

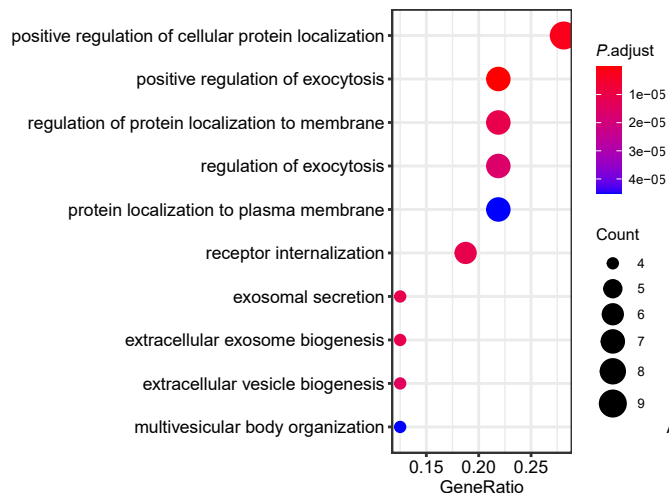

B

## KEGG Pathway Enrichment

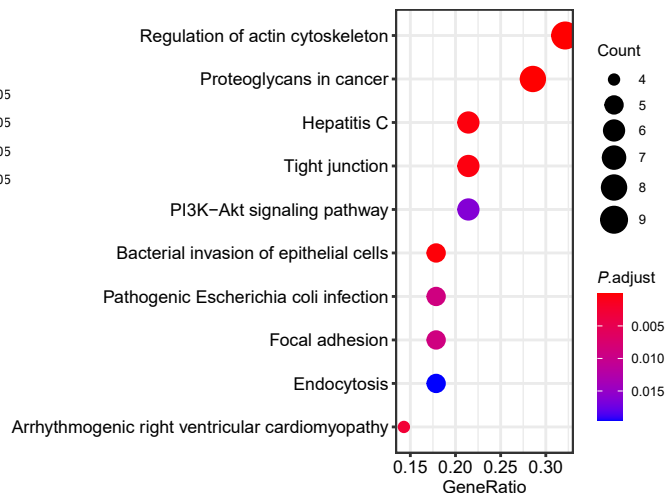

Supplement: Supplementary file 1 — Additional file 1: Figure S1. TEXscore is associated with exosome features. (A) The top ten signaling pathways enriched of TEXscore genes by Gene ontology (GO) analysis were shown. (B) The top ten signaling pathways enriched of TEXscore genes by KEGG analysis were shown. [file 12967_2021_3053_MOESM1_ESM.pdf]

A

TCGA-BRCA

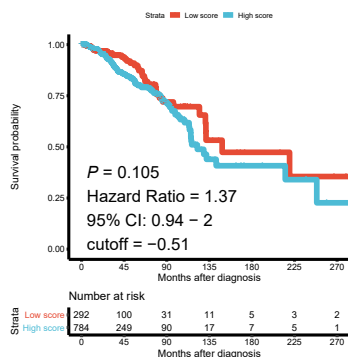

B

TCGA-CESC

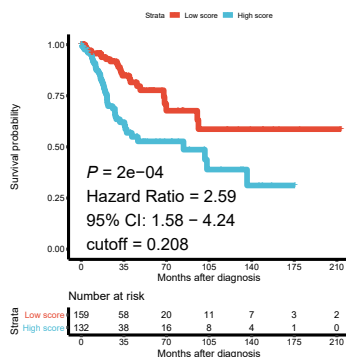

C

TCGA-GBM

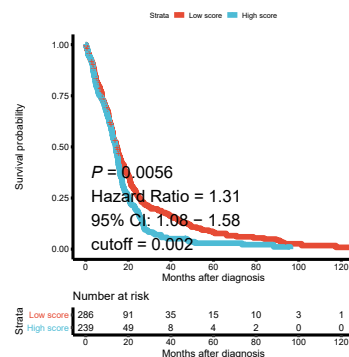

D

TCGA-HNSC

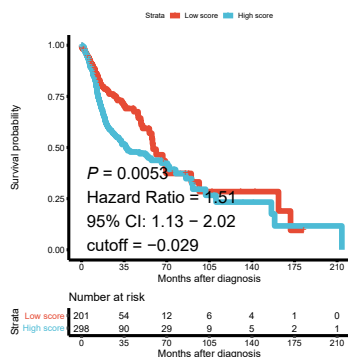

E

TCGA-OV

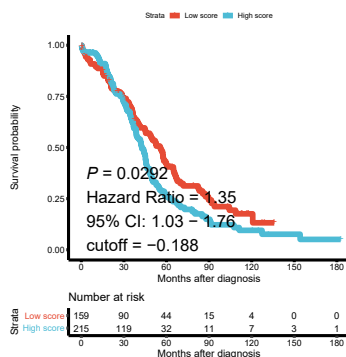

F

TCGA-PRAD

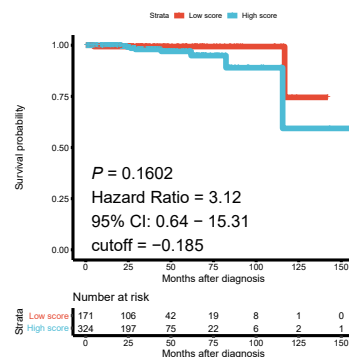

G

TCGA-SKCM

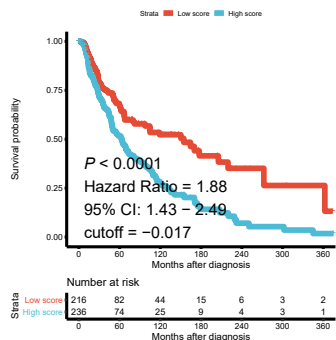

H

TCGA-UCEC

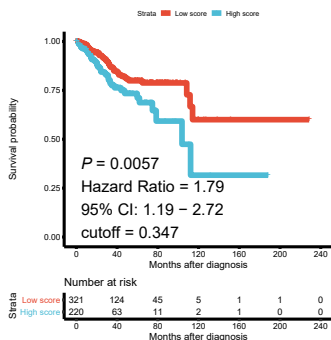

I

GSE62254

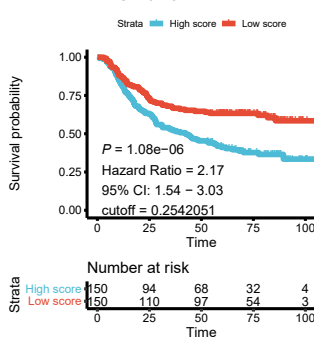

J

GSE30219

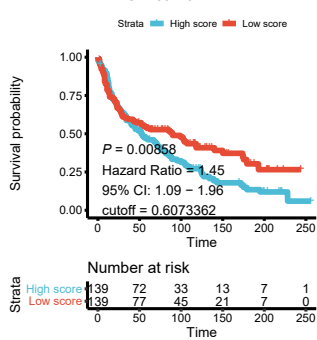

Supplement: Supplementary file 2 — Additional file 2: Figure S2. High TEXscore correlated with poor overall survival. (A-J) Kaplan‐Meier survival curves suggested that a poor overall survival of patients with high TEXscore (high: blue; low: red) in (A) TCGA-BRCA dataset (P = 0.105, Hazard ratio = 1.37, 95%CI = 0.94–2), (B) TCGA-CESC dataset (P = 2e-04, Hazard ratio = 2.59, 95%CI = 1.58–4.24), (C) TCGA-GBM dataset (P = 0.0056, Hazard ratio = 1.31, 95%CI = 1.08–1.58), (D) TCGA-HNSC dataset (P = 0.0053, Hazard ratio = 1.51, 95%CI = 1.13–2.02), (E) TCGA-OV dataset (P = 0.0292, Hazard ratio = 1.35, 95%CI = 1.03–1.76), (F) TCGA-PRAD dataset (P = 0.1602, Hazard ratio = 3.12, 95%CI = 0.64–15.31), (G) TCGA-SKCM dataset (P < 0.0001, Hazard ratio = 1.88, 95%CI = 1.43–2.49), (H) TCGA-UCEC dataset (P = 0.0057, Hazard ratio = 1.79, 95%CI = 1.19–2.72), (I) GSE62254 dataset (P = 1.08e-06, Hazard ratio = 2.17, 95%CI = 1.54–3.03), (J) GSE30219 dataset (P = 0.00858, Hazard ratio = 1.45, 95%CI = 1.09–1.96). [file 12967_2021_3053_MOESM2_ESM.pdf]

A

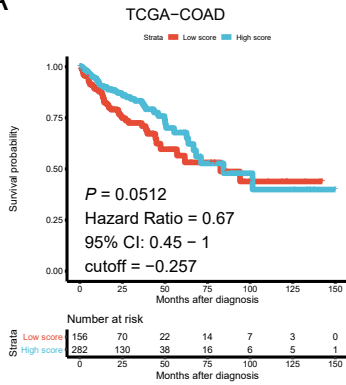

B

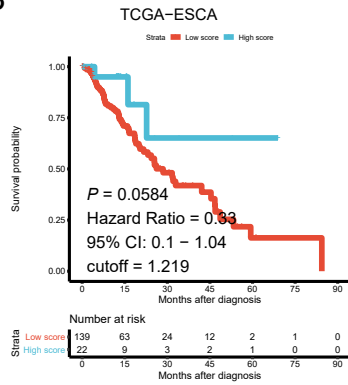

C

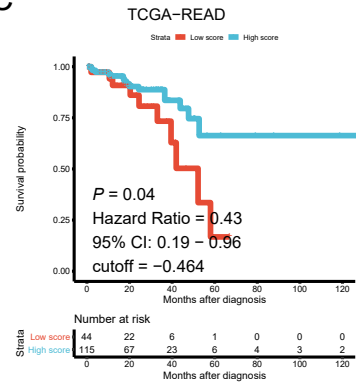

D

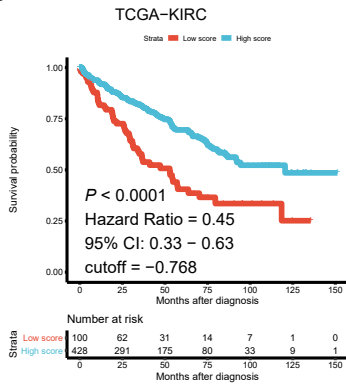

E

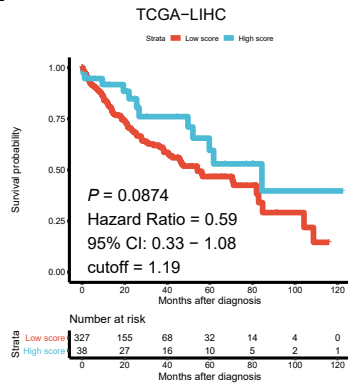

Supplement: Supplementary file 3 — Additional file 3: Figure S3. High TEXscore correlated with favorable overall survival. (A-E) Kaplan‐Meier survival curves suggested that a better overall survival of patients with high TEXscore (high: blue; low: red) in (A) TCGA-COAD dataset (P = 0.0512, Hazard ratio = 0.67, 95%CI = 0.45–1), (B) TCGA-ESCA dataset (P = 0.0584, Hazard ratio = 0.33, 95%CI = 0.1–1.04), (C) TCGA-READ dataset (P = 0.04, Hazard ratio = 0.43, 95%CI = 0.19–0.96), (D) TCGA-KIRC dataset (P < 0.0001, Hazard ratio = 0.45, 95%CI = 0.33–0.63), (E) TCGA-LIHC dataset (P = 0.0874, Hazard ratio = 0.59, 95%CI = 0.33–1.08). [file 12967_2021_3053_MOESM3_ESM.pdf]

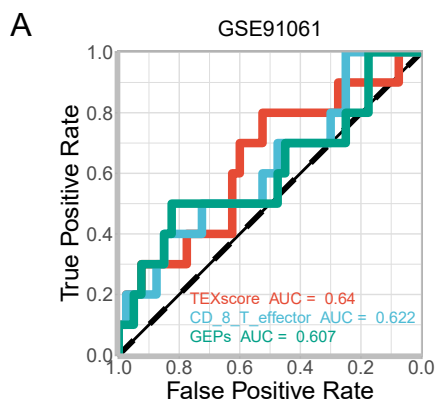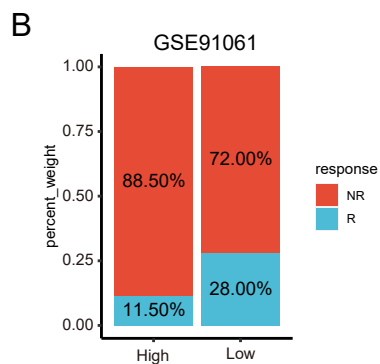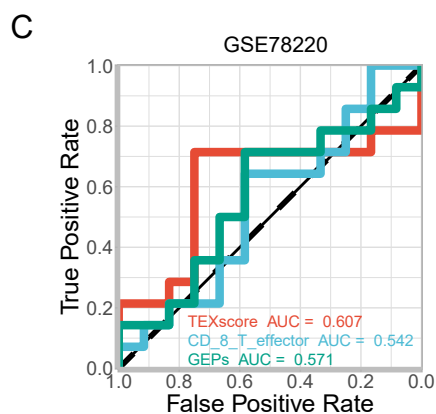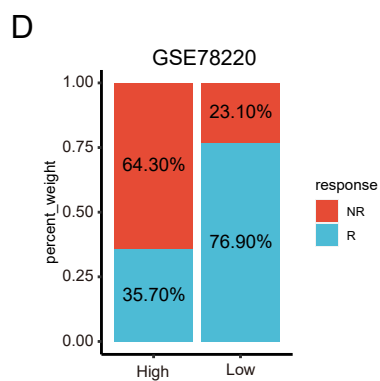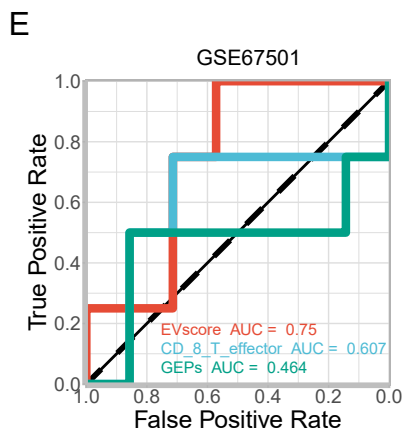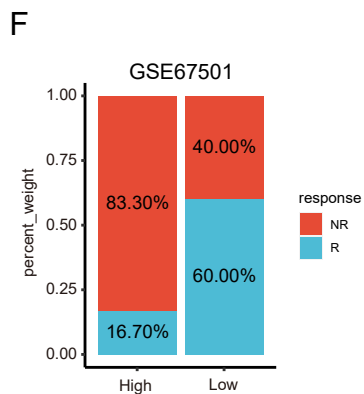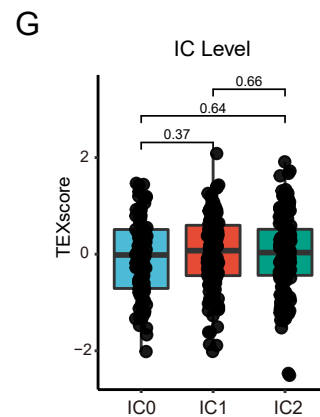

Supplement: Supplementary file 4 — Additional file 4: Figure S4. Predictive value of TEXscore towards immune checkpoint blockers in independent cohorts. (A) ROC curve suggested that TEXscore exerted inferior predictive capacity to ICB response in GSE91061 cohort. (TEXscore: AUC = 0.64, CD8+ T cells: AUC = 0.622, GEP: AUC = 0.607). (B) Rate of clinical response (non-response (NR) and response (R)) to ICB in high or low TEXscore groups in the GSE91061 cohort. (C) ROC curve suggested that TEXscore exerted inferior predictive capacity to ICB response in GSE78220 cohort. (TEXscore: AUC = 0.607, CD8 + T cells: AUC = 0.542, GEP: AUC = 0.571). (D) Rate of clinical response (NR and R) to ICB in high or low TEXscore groups in the GSE78220 cohort. (E) ROC curve suggested that TEXscore exerted inferior predictive capacity to ICB response in GSE67501 cohort. (TEXscore: AUC = 0.75, CD8 + T cells: AUC = 0.607, GEP: AUC = 0.464). (F) Rate of clinical response (NR and R) to ICB in high or low TEXscore groups in the GSE67501 cohort. (G) IC level represented PD-L1 expression on tumour-infiltrating immune cells through immunohistochemistry (IHC). IC level was not related to the TEXscore. Specimens were scored as IHC IC0, IC1, or IC2 if < 1%, ≥ 1% but < 5%, or ≥ 5% of IC were PD-L1 positive, respectively. [file 12967_2021_3053_MOESM4_ESM.pdf]

A

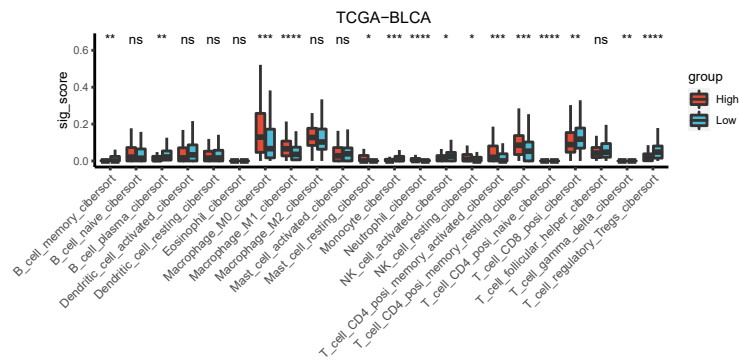

B

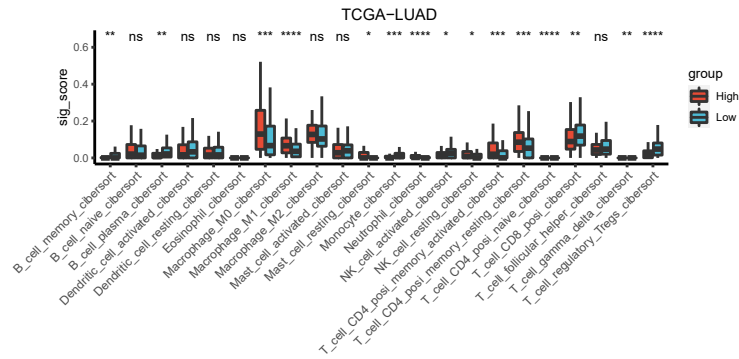

C

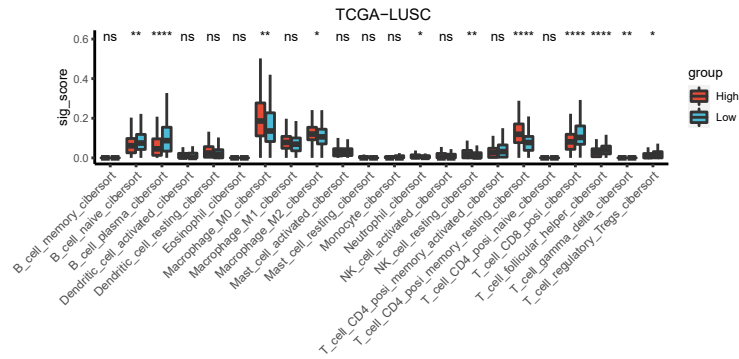

D

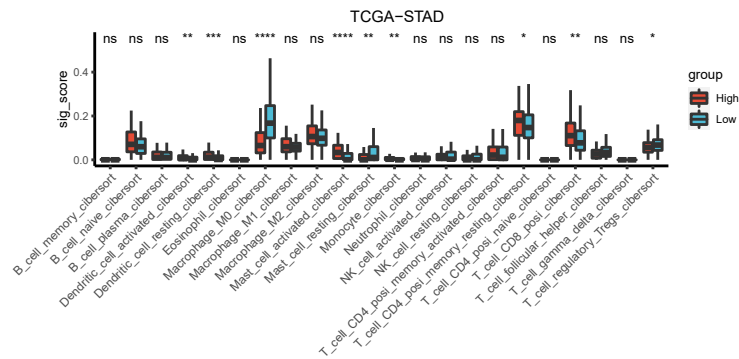

Supplement: Supplementary file 5 — Additional file 5: Figure S5. Immune cell infiltration level in high and low TEXscore settings validated by CIBERSORT in TCGA. (A-D) High TEXscore was accompanied with alteration of M0 and M2 macrophage infiltration in (A) TCGA-BLCA cohort, (B) TCGA-LUAD cohort, (C) TCGA-LUSC cohort, (D) TCGA-STAD cohort. [file 12967_2021_3053_MOESM5_ESM.pdf]

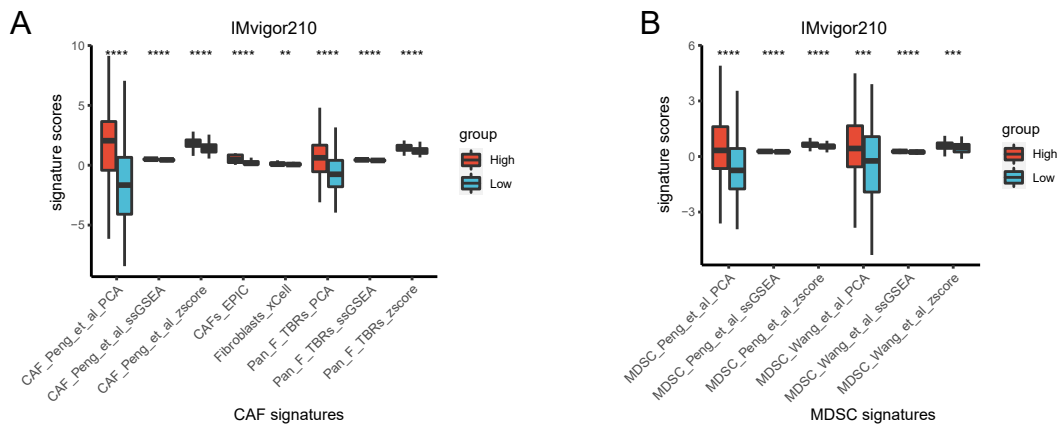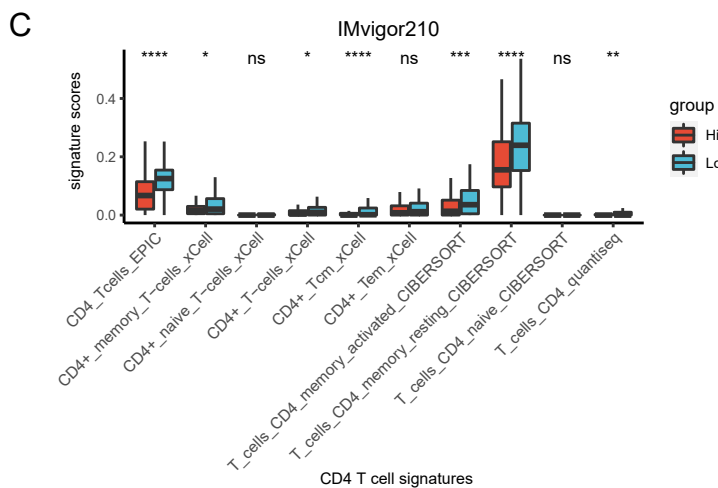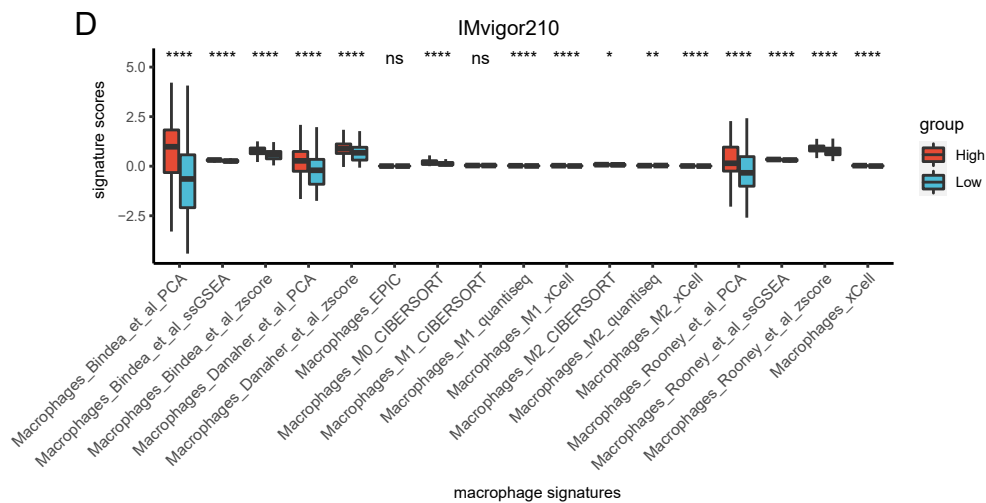

Supplement: Supplementary file 6 — Additional file 6: Figure S6. Tumor microenvironment signatures in IMvigor210 dataset. (A) Expression of CAFs associated signatures elevated in high (red) TEXscore versus the low (blue) in IMvigor210. (B) Expression of MDSC associated signatures elevated in high (red) TEXscore versus the low (blue) in IMvigor210. (C) Expression of CD4 + T cell associated signatures elevated in low (blue) TEXscore versus the high (red) in IMvigor210. (D) Expression of macrophage associated signatures elevated in high (red) TEXscore versus the low (blue) in IMvigor210. P-values are shown with ****, ***, **, *, ns representing P < 0.0001, P < 0.001, P < 0.01, P < 0.05, no significant, respectively. [file 12967_2021_3053_MOESM6_ESM.pdf]
